# Nicotine: Sporting Friend or Foe? A Review of Athlete Use, Performance Consequences and Other Considerations

**DOI:** 10.1007/s40279-017-0764-5

**Published:** 2017-08-08

**Authors:** Toby Mündel

**Affiliations:** School of Sport and Exercise, Massey University, Private Bag 11 222, Palmerston North, 4442 New Zealand

## Abstract

Nicotine use amongst athletes is high and increasing, especially in team sports. This narrative review examines the rationale behind its use and evidence of its effect on physical performance, and considers important factors that should determine future research efforts. To date, ten studies have assessed muscular strength and power, sub- or maximal endurance and high-intensity exercise when nicotine (medication) or smokeless tobacco was used as an intervention. Two studies observed an ergogenic effect, one an ergolytic with the remaining seven reporting no change. These studies have notable limitations and confounding factors that include participant tolerance to nicotine, interindividual responses, the nicotine delivery system used and failure to adhere to rigorous experimental/scientific design. Further research is encouraged to address these limitations and determine the extent to which anti-doping and governing bodies should consider promoting, coordinating and monitoring any effort against nicotine and nicotine-containing substances in sports.

## Key Points

**Table:** 

At present, the use of nicotine is not prohibited by the World Anti-Doping Agency.
Nicotine is available over-the-counter and use is widespread amongst professional team/strength sports (e.g. American football, ice hockey, wrestling, bobsleigh, gymnastics, rugby, skiing) whereby active consumption of nicotine and nicotine-containing substances in-competition occurs in approximately 25–50% of such athletes.
Nicotine’s mode of action includes both psychostimulatory and sympathomimetic effects.
Of the 16 performance investigations presented in the ten studies reported herein involving nicotine or a nicotine-containing substance to date, the majority (12) have demonstrated no significant effect. However, the current evidence base is limited both in the quantity and quality of studies performed.
This literature review outlines important considerations for a more complete interpretation of results, and proposes future avenues of investigation that should determine the true magnitude of whether nicotine enhances performance, increases the health risk to an athlete and alters the spirit of sport.

## Introduction

Together with caffeine, nicotine is considered the most widely consumed psychoactive substance in the world [10]. Consumption patterns include smoking (cigarettes, cigars, pipes) and smokeless (*chewing tobacco* that creates salivation and the need for spitting, a moist form of chewing tobacco called *snus* placed under the lip without the need for spitting, and a dry powdered form of *snuff* inhaled through the nose) tobacco as well as nicotine replacement therapies (NRTs) marketed in gum, transdermal patches, nasal sprays, inhalers, lozenges, tablets, etc. Nicotine naturally occurs in some edible plants such as cauliflower, aubergines, potato and tomato; however, in much smaller concentrations and, therefore, is unlikely to contribute meaningfully even if unrealistically large volumes were to be consumed [17]. In the sporting domain, use of nicotine, in the forms of chewing tobacco and (moist) snuff became popularised amongst professional baseball players in the late 1970s and 1980s, and in the last decade its use has been discovered especially in the winter sports. This led the World Anti-Doping Agency (WADA) to place nicotine on its Monitoring Program in 2012 [59], indicating that it wished to detect patterns of misuse in order to determine whether it satisfied the three criteria of enhancing sport performance, being a potential health threat to an athlete and altering the spirit of sport that could yet see it upgraded to the List of Prohibited Substances.

When used, nicotine exerts psychological and physiological effects that should, based on research findings, be nootropic and ergogenic. Yet what is the evidence base that might support or oppose the use of nicotine in the athletic arena? There have been reviews published that have included sub-sections on nicotine and human performance [49], been focussed on the cardiovascular response to smokeless tobacco (ST [11]) and physiological exercise responses to tobacco smoking [29], focussed on nicotine-naïve individuals [33] or on attention and cognition as outcome measures [25]. No attempt has been made to review nicotine’s potential for influencing physical performance that would be of interest to the sporting community.

The purpose of this review is to document nicotine use amongst athletes and across sports, briefly discuss the pharmacology and mechanisms of action of the drug, review those studies that have performance as a measure, and then consider important issues that should dictate future research efforts. This narrative review includes assessment of quality-assured, peer-reviewed publications available through to the end of June 2017 primarily using the PubMed database and Google Scholar search engine, whilst second- and third-order reference lists were checked manually for relevant articles. Studies were excluded when interventions exclusively involved tobacco smoking or smokers; the rationale for this was that (i) tobacco use, primarily through smoking, is the leading cause of preventable death and kills approximately 6 million people globally each year as reported by the World Health Organization (WHO [60]); (ii) tobacco has been demonstrated to reduce aerobic and muscular performance [14, 28, 61], and (iii) elite/professional athletes report considerably lower smoking prevalence (0.8–15%) than controls (17–28%) and the general population worldwide (~25%) [1, 13, 41, 56, 60].

## Prevalence of Use Amongst Athletes and Sports

### Cross-Sectional, Self-Report Studies

According to Connolly et al. [13], ST has been associated with baseball since its inception, and despite being replaced by smoking in the 1950s, returned in the late 1970s. Reports of up to a third of collegiate baseball and football players using ST have surfaced [12, 44], and this was substantiated by 34% of major league baseball players being current users [13]. By 2003, the number of major league players regularly using ST was 36% [56]. Perhaps surprisingly, there have been reports from Finland [42] and Switzerland [27] that a dose-response relationship exists between ST and physical activity, such that ST use is markedly increased amongst those reporting greater frequency and intensity of sport/exercise, especially regularly competitive, and with increased physical fitness. Furthermore, athletes in team sports, ice-hockey and resistance exercise were associated with the greatest use of ST [42]. The use of snus amongst Norwegian adolescent elite athletes [41] was 17%, although more common amongst team sport (21%) than individual athletes (10%). Of the 446 elite Finnish athletes funded by their National Olympic Committee in 2002, prevalence of daily snuff use was fivefold that of controls at 25%, being highest for team sports and lowest for endurance athletes [1]. Of course the design of these cross-sectional studies precludes causal conclusions and they are limited by self-report data (over-/underestimation). However, potential bias due to selective response is unlikely as response rates were high (79–95%), and several of the studies described [1, 27, 42] report good validity of self-report tobacco use in their populations.

### Anti-Doping Urine Sample Analysis

As part of the regular doping control procedure during the 2009 men’s World Ice-Hockey Championships in Switzerland, urine samples of two players from every team were collected after games (*n* = 72). Traces of nicotine or any four of its major metabolites were present in every sample, suggesting environmental exposure or active consumption [38]. However, allowing for the lower limit of quantification and hypothesizing a conservative concentration limit for active consumption of 50 ng ml^−1^, the authors determined that active nicotine use was evident in at least 36% (but at least one of nicotine’s metabolites was present in 53%) of samples that would have meant nicotine use close to and/or during the game due to nicotine’s short half-life. Further, two samples presented nicotine concentrations exceeding the upper limit of quantification, with such acute exposure to nicotine hardly achievable for even a regular consumer [9], and as such increasing the likelihood of use for doping. Following this, Marclay et al. [39] reported on a 1-year monitoring study of 2185 urine samples from professional athletes in 43 different sport disciplines—every specimen that was analysed by the Swiss Laboratory for Doping Analyses. Traces of nicotine or any four of its major metabolites were detected in 23% of the samples, but using the same conservative limit for active consumption as described above, prevalence of ‘active’ nicotine consumption immediately prior to and/or during sport practice was determined at 15% [39]. Of note, cumulative exposure of >25% (*greater* than the worldwide prevalence of smoking in the general population, as reported by the WHO) was reported in American football (56%), ice hockey (32%), wrestling (32%), bobsleigh (31%), gymnastics (29%), rugby (28%) and skiing (26%).

Therefore, from the limited evidence currently available it can be concluded that use of nicotine is high within the elite and professional athlete environment. Sports with the most use include baseball and ice-hockey but other strength-based and winter sports see high use, with most studies confirming team sport athletes using nicotine the most, and endurance athletes the least. Although the administration of nicotine is likely via ST, current analyses of biological samples are not able to distinguish between sources.

## Rationale for Use

Nicotine, 3-(1-methyl-2-pyrrolidinyl)pyridine, is the principle alkaloid commonly found in the nightshade family of plants, but perhaps most often associated with cultivated tobacco where it constitutes ≤3% of the dry weight. Having natural host-plant resistance, nicotine was used extensively as an insecticide, although more recently neonicotinoids have replaced its use. Recreationally, nicotine is consumed as a stimulant through tobacco consumption, whilst ironically (or perversely) the primary therapeutic use of nicotine is to treat nicotine dependence from smoking tobacco to improve health outcomes.

### Why Athletes Report They Consume Nicotine/Nicotine-Containing Substances

Athletes’ own beliefs are that consumption of nicotine/nicotine-containing substances (i.e. ST) proves ergogenic by preventing xerostomia (dry mouth [12]) as saliva secretion is augmented, controlling weight [3] as satiety is stimulated, improving reaction time and concentration [23], and helping relaxation and desirable arousal-attention [13]. These latter reports are substantiated by a meta-analysis determining that the stimulant effects of nicotine enhance aspects of cognition and attention, namely motor abilities, attention and memory [25].

### Brief Pharmacology of Nicotine and Mechanisms of Action

For excellent and more detailed reviews on the human pharmacology of nicotine, readers are directed towards Hukkanen et al. [31] and Benowitz et al. [7].

Depending on the product/vehicle, concentrations of nicotine vary to a reasonable extent, as do systemic absorption and bioavailability (Table 1) and the resultant pharmacological effects. Absorption of nicotine across biological membranes is dependent on pH. When acidic, such as with smoking tobacco, nicotine is primarily ionised, and as a consequence little buccal absorption occurs; however, ST and NRT are usually buffered to alkaline to facilitate buccal or other membrane absorption as considerable nicotine is unionised [7]. Following absorption nicotine is quickly (within seconds) and extensively distributed through the bloodstream reaching peak concentrations in 30–60 min, and readily crosses the blood-brain barrier to exert its effects [7]. Absorption of nicotine is slower and the increase in systemic levels more gradual from NRT than from tobacco sources (Table 1). Tissues with the highest affinity for nicotine include the liver, kidney, spleen, lung and brain [7]. The elimination half-life of nicotine within the circulation is 1–2 h and it is primarily metabolised by hepatic cytochrome P450 enzymes [7]. Quantitatively (70–80%) the most important metabolite of nicotine is cotinine, and as its metabolism is much slower than that of nicotine a longer half-life (~16 h) and reduced daily fluctuation favours it as a biomarker for nicotine intake, especially in urine samples [7]. There is considerable interindividual variability in nicotine metabolism (e.g. nutrition, age, sex, medication, ethnicity, genetics, etc.), although pertinent factors will be discussed in Sect. 4.5.

**Table 1:** Different forms of nicotine administration and absorption pharmacokinetics. Adapted from Hukkanen et al. [31], with permission

Nicotine administration and dose	*C*_max_ (ng ml^−1^)	*T*_max_ (min)	Bioavailability (%)	References
Smoking tobacco (one cigarette, 5 min, ~2 mg/cigarette)	15–30	5–8	80–90	Benowitz et al. [7]
Smokeless tobacco (1 g Swedish snus, 60 min, ~11 mg/portion)	11	60	24–32	Digard et al. [16]
Gum (4 mg in gum, 30 min)	9	45	63	Digard et al. [16]
Transdermal patch (one daytime patch, 15 mg/16 h)	11–14	6–9 h	75–100	Benowitz et al. [7]
Inhaler (one 10 mg cartridge, 20 min)	8	30	51–56	Benowitz et al. [7]
Sublingual tablet (2 mg, 20–30 min)	4	60	65	Benowitz et al. [7]
E-cigarette ‘vaping’ (65 min puffing, 18 mg ml^−1^)	14–16	70–75	–	Marsot and Simon [40]

All values are for venous blood

*C*_*max*_ peak blood concentration, *T*_*max*_ time to peak blood concentration

Nicotine is structurally similar to acetylcholine and is a nicotinic acetylcholine receptor (nAChR) agonist. Nicotine elicits its psychoactive effects by activating neuromediators centrally (e.g. dopamine, serotonin, the catecholamines, etc.). Nicotine administration leads to increased dopamine cell firing in the ventral tegmental area [15] and increased dopamine release in the nucleus accumbens [52], actions which are thought to be critical to the properties of nicotine as an addictive drug. This mesolimbic pathway in response to low-dose nicotine exerts psychostimulatory effects, whilst at high doses a depressant or relaxant effect occurs through activation of endogenous opioid pathways [57]. These actions are responsible for the reinforcing effects of nicotine that usually occur in the absence of euphoria.

Peripherally, the sympathoadrenal effects of nicotine are well known, especially those pertaining to acute cardiovascular consequences [24]. For example, in healthy humans nicotine per se increases heart rate and blood pressures [6, 50, 54], and causes cutaneous vasoconstriction or decreased skin temperature [6, 54], systemic venoconstriction [19], and increased muscle blood flow [55], and has been shown to alter coronary haemodynamics and O_2_ transport [34].

It has also been experimentally demonstrated that nicotine exerts antinociceptive effects by increasing the pain threshold independent from the putative mood effects of nicotine [32].

In summary, circulating levels of nicotine resulting from ST or NRT use result in agonism of cholinergic receptors in the central nervous system (CNS) and peripheral tissues, especially reward pathways in the brain and the splanchnic release of adrenomedullary hormones. Peak concentrations and metabolism of nicotine occur rapidly, hence the use of nicotine’s primary metabolite, cotinine, as a biomarker.

## Review of Physical Performance Studies

This section reviews human studies concerning the effect of nicotine on physical performance with the exception of those activities where performance is measured by psychomotor (gross or fine) abilities, e.g. reaction/movement times. This methodology was used in some of the earlier baseball-oriented research (e.g. Edwards et al. [20] and Landers et al. [35]) and is included in an excellent meta-analysis by Heishman et al. [25], whereas the focus here was of outcome measures utilising a larger muscle mass, preferably whole-body, that can be easily applied to most athletic events. Tables 2 and 3 display details of the methodology and primary outcome measure(s) for ST and NRT, respectively, whereas the following discussion is subdivided into type of athletic activity, namely (i) muscular performance (strength and power), (ii) submaximal and maximal aerobic endurance, and (iii) high-intensity exercise and intermittent sprints.

**Table 2:** Studies investigating the effect of smokeless tobacco on exercise performance

Study	Participants	Performance protocol	Tobacco intervention	Performance effect
Baldini et al. [3]	12 habitual smokeless tobacco users abstinent ≥10 h, and 6 non-users as controls	30-s Wingate test with a resistance of 7.5% bodyweight	20 min smokeless tobacco use whilst resting supine, then tobacco discarded and performed Wingate test. Four conditions: zero, 1/3 mean, mean, mean +2/3 where ‘mean’ refers to individual average of 10 dips	5-s peak and 30-s mean power output unaffected by dose and similar to controls
Van Duser and Raven [58]	15 habitual smokeless tobacco users abstinent ~12 h	Graded maximal treadmill test at 93.8 m min^−1^ with incline increasing 2.5% min^−1^ to volitional exhaustion	30 min smokeless tobacco use whilst resting seated, then tobacco discarded and performed maximal protocol. 2.5 g smokeless tobacco compared to placebo chew	Not reported but maximal O_2_ uptake and lactate unchanged
Escher et al. [21]	20 habitual smokeless tobacco users, and 20 non-users as controls	Maximum voluntary force and rate of force development using knee extension at 250° s^−1^ from 90° to 0° of flexion	2 conditions for habitual users: (1) abstinent ≥12 h and remained abstinent for testing, (2) one dip 2 h prior to and then again on arrival at the laboratory whilst testing	Maximal force (12%) and rate of force development (9–10%) reduced when using smokeless tobacco compared to abstinent
Morente-Sánchez et al. [45]	18 non-users/non-smokers	Maximum handgrip strength, maximum countermovement jump height, agility shuttle run, Yo-yo recovery intermittent shuttle test (level 1)	40 min smokeless tobacco use whilst resting supine, then handgrip strength, countermovement jump and agility shuttle run were performed before tobacco discarded after 70 min and the Yo-yo test was performed. 1.0 g smokeless tobacco compared to placebo chew	All performance tests were unaffected by smokeless tobacco
Zandonai et al. [62]	14 non-users/non-smokers	Cycle to volitional exhaustion at 65% maximal aerobic power	Smokeless tobacco use from start of exercise and throughout exhaustion trial. 1.0 g smokeless tobacco compared to placebo chew	Endurance time unaffected with smokeless tobacco

**Table 3:** Studies investigating the effect of nicotine on exercise performance

Study	Participants	Performance protocol	Nicotine intervention	Performance effect
Mündel and Jones [46]	12 non-smoker, healthy, active males	Cycle to volitional exhaustion at 65% maximal aerobic power	7 mg (24 h^−1^) transdermal nicotine patch applied to deltoid muscle the evening prior to testing i.e. overnight (≥10 h treatment). Compared to placebo patch	Endurance time improved (17%) with nicotine
Pyšný et al. [53]	11 male and 7 female, healthy, active non-smokers	Two 30-s Wingate tests separated by 30 min, each with a resistance of 0.106 W kg^−1^ (male) and 0.089 W kg^−1^ (female)	4 mg sublingual nicotine tablet use whilst resting supine 20 min prior to second Wingate. Compared to placebo tablet	All performance parameters were unaffected by nicotine
Mündel et al. [47]	9 non-smoker, healthy, active males	Maximum leg extensor torque (concentric, eccentric and isometric), maximum countermovement jump height, 30-s Wingate test with a resistance of 7.5% bodyweight	2 and 4 mg nicotine chewing gum use whilst resting seated for 20 min then discarded before tests began. Compared to placebo gum	Peak torque (concentric, eccentric and isometric) improved (6%) with 2 mg nicotine, countermovement jump and Wingate performance unaffected by nicotine
Fogt et al. [22]	10 male and 10 female, healthy non-smokers	Incremental maximal cycle test starting at 15 W with work-rate increasing 15 W min^−1^ to volitional exhaustion	Vaporiser nicotine (18 mg cartridge) inhaled once every 30 sec for 10 min via electronic cigarette whilst resting seated, 55 min prior to maximal test. Compared to placebo vaporiser inhalation (0 mg cartridge)	Maximal aerobic performance unaffected by nicotine
Druyan et al. [18]	16 healthy, active, males: 8 smokers abstinent ~12 h and 8 non-smokers	Graded maximal treadmill test at fixed unknown speed with incline increasing 2% every 2 min to volitional exhaustion	2 mg nicotine lozenge for 10 min followed by maximal protocol. Compared to control (no nicotine)	Not reported but maximal O_2_ uptake and anaerobic threshold unchanged

### Characteristics of Studies

Overall ten published studies have used protocols that have yielded 16 separate measures of performance; three studies assessed muscular strength and power, five assessed (sub/maximal) endurance, whilst four assessed high-intensity exercise. Five studies used ST (moist tobacco ‘dip’) as an intervention and five nicotine, each with a different delivery form (transdermal, sublingual tablet, chewing gum, vaporiser and lozenge). A majority (seven) of studies used males only whilst none exclusively used females. The average sample size of the nine studies was 17 (SD = 9, median = 16, range = 9–40). All study samples reported young (mean age ≤25 years) and healthy participants, at least recreationally and competitively active but not at (sub-)elite level. Seven studies included nicotine-naïve participants, whilst of the four studies that included habitual nicotine users three were conducted under nicotine abstinence of ≥10 h with the fourth study including both abstinent (≥12 h) and user conditions. A majority (seven) of studies were placebo-controlled whereas the remaining three simply used a control condition where no intervention was present. Six of the studies performed a manipulation check by measuring blood/urine cotinine (six) and/or blood nicotine (three) concentrations.

### Effects on Muscular Strength and Power

Of the three studies that assessed muscular strength and power, one showed an ergogenic effect with nicotine [47], one an ergolytic effect [21] and another no change [45]. Escher et al. [21] tested college athletes who were regular ST users following ~12 h abstention from tobacco or having consumed ST 2 h and immediately prior to testing. They reported that leg extensor force and rate of force development was lower when using compared to abstaining from tobacco. Morente-Sánchez et al. [45] assessed handgrip strength and countermovement jump height following 40 min snus or placebo use in amateur footballers who were not habitual tobacco users, and reported that snus use did not affect either performance measure. Mündel et al. [47] tested leg extensor force and countermovement jump height in nicotine-naïve team sport players when chewing placebo or 2 or 4 mg nicotine gum. Results showed that leg extensor torque was improved with the low-dose nicotine compared to the placebo only but that neither nicotine gum influenced countermovement jump height.

### Effects on Submaximal and Maximal Endurance

Of the five studies that assessed sub- or maximal endurance, only one demonstrated an ergogenic effect with nicotine [46], whilst the other four demonstrated no change [22, 62] or did not explicitly report time or work completed [18, 58]. Mündel and Jones [46] had nicotine-naïve participants cycle to exhaustion at ~75% of their aerobic maximum following ~10 h of transdermal nicotine treatment or placebo, and reported a significant improvement in endurance with nicotine. Zandonai et al. [62] also used nicotine-naïve participants cycling to exhaustion at ~65% of their aerobic power as ST or a placebo was placed into the mouth at the beginning of exercise and was kept there until exhaustion, and reported no effect on endurance with ST. Fogt et al. [22] performed an incremental maximal cycle test on non-smokers following vaporised nicotine or placebo delivery and reported no effect on peak power achieved. Van Duser and Raven [58] and Druyan et al. [18] performed graded maximal treadmill tests on regular tobacco users who were ~12 h abstinent, although Druyan et al. [18] also included a non-user group. Van Duser and Raven [58] reported no differences in aerobic capacity or peak lactate concentration between ST and placebo use for ~30 min pre-exercise. Similarly, Druyan et al. [18] reported no differences in aerobic capacity or anaerobic threshold when a 2-mg nicotine lozenge was used for 10 min pre-exercise compared to a control condition for both their smoker and non-smoker groups. Although neither studies describe the performance component/results of their graded maximal treadmill tests, the authors confirmed that distance/work completed was also similar between conditions (personal communication).

### Effects on High-Intensity Exercise

Of the four studies that assessed high-intensity and intermittent sprint exercise, only one demonstrated an ergogenic effect with nicotine [47] whilst the other three demonstrated no change [3, 45, 53]. Three studies have assessed anaerobic performance using the 30-s Wingate test; Baldini et al. [3] tested regular ST users ~10 h abstinent under different amounts/doses of ST, whilst Pyšný et al. [53] and Mündel et al. [47] tested nicotine-naïve participants who had consumed a 4-mg sublingual tablet or 2 and 4 mg gum, respectively, compared to a placebo. Baldini et al. [3] reported no change in peak or average power, Pyšný et al. [53] noted no differences between nicotine and placebo whilst Mündel et al. [47] demonstrated that neither nicotine gum affected anaerobic capacity but that low-dose (2 mg) gum did affect the pacing strategy adopted. Morente-Sánchez et al. [45] used maximum agility and progressive shuttle running tests following 40 min snus or placebo use in amateur footballers who were not habitual tobacco users, and reported that snus use did not affect either performance test.

### Effects on Physical Performance: Summary and Considerations

Of the ten published studies, 16 separate performance measures/tests have been reported, of which a majority (12) have demonstrated nicotine to have no effect, though two have shown an ergogenic effect and two an ergolytic effect. Therefore, the evidence base reviewed suggests that descriptively nicotine is unlikely to affect performance but if it does then it is equally likely to be beneficial as detrimental. However, there are several important considerations that need to be raised in order to critically determine the likely true magnitude and direction of nicotine’s effect.

#### Tolerance and Sensitivity

An individual’s sensitivity and/or tolerance to nicotine need to be acknowledged, whereby regular/chronic users display dampened responses to the same nicotine dose compared to naïve/acute users [50]. Regular nicotine users who abstain develop withdrawal symptoms (craving, irritability, depression, restlessness, hunger, difficulty concentrating and consequent performance decline) that occur as early as 30 min following deprivation [26] and can last 24 h [48]. Only five of the performance studies report data on side effects [22, 45–47, 62]; all used nicotine-naïve participants who reported either minor (occasional coughing/sneezing, scratchy/tickly throat) or major (tachycardia, nausea, dizziness, etc.) adverse events that even led to participants suspending trials. It is noteworthy, however, that none of these studies demonstrated an ergolytic effect of nicotine, although two observed ergogenic effects. Furthermore, of the four studies that included habitual nicotine users [3, 18, 21, 58], only one [21] noted an ergolytic effect when regular users *consumed* rather than *abstained* from tobacco, whilst the other three demonstrated no such performance detriment with nicotine use when users remained abstinent. Therefore, nicotine can display a biphasic effect at the neuromuscular junction whereby it exerts excitatory (acutely, when naïve or after withdrawal) then inhibitory (chronically, when tolerant) effects [2, 37].

#### Individual Response

Another salient point concerns the considerable interindividual variability in nicotine metabolism [6], of which genetic variations are discussed elsewhere [31]. Given the high degree of hepatic extraction, any physiological event that perturbs/reduces liver blood flow (e.g. meals, posture, exercise, etc.) will likely affect the rate of nicotine clearance and, therefore, reduce and/or delay the maximal effect observed [7]. Consumption of menthol or grapefruit will likely inhibit cytochrome P450 2A6, the primary enzyme responsible for the oxidation of nicotine and cotinine [7]. Age—at least in adults—does not appear to affect clearance until >65 years; however, women metabolise nicotine faster than men and this is further accelerated in those taking oestrogen-containing oral contraception [8], though menstrual cycle phase has no effect [30]. Ethnic differences in nicotine metabolism have also been observed with African- and Chinese-Americans exhibiting slower clearance than Hispanic and Caucasian Americans [7].

#### Delivery System

Nicotine can be delivered via several different routes using different products. As this results in different nicotine bioavailability and pharmacokinetics (Table 1), it is important to consider this for appropriate study design and correct interpretation of results. For example, buccal administration (i.e. gum, inhaler or tablet) often results in lower (peak) systemic concentrations than do transdermal and tobacco sources, and participants need to be instructed on the correct use to maximise buccal absorption and minimise loss due to swallowing and subsequent first-pass metabolism. However, these products are also conveniently available as zero or non-nicotine placebos and, as such, are a valuable tool for acute interventions. Another consideration with NRT, as with many over-the-counter products (especially pharmacological), concerns whether a fixed dose should be administered as opposed to individualising a dose (e.g. to body mass). This needs to be more carefully considered for future studies as all previous NRT studies [18, 22, 46, 47, 53] have administered fixed doses. However, interestingly and consistent with the known pharmacological action of low-dose nicotine as a stimulant and higher-dose nicotine as a depressant [36, 57], this dose-response relationship is reflected by performance responses whereby lower doses prove nootropic/ergogenic whilst higher doses do not [47, 50, 51].

#### Consequences on Health and Disease

It would be remiss to omit any discussion concerning the health (disease) consequences of nicotine consumption. Although nicotine is not the direct cause of most tobacco-related diseases, its addictiveness sustains continued tobacco product use and the associated exposure to an array of bioactive compounds and carcinogens contributes to tobacco use being the leading cause of preventable death globally each year [31, 60]. However, the focus of this sub-section is not on *tobacco* but only briefly to highlight how *nicotine* use per se contributes to health and disease from an athlete’s perspective; therefore readers are directed to more comprehensive critique and discussion elsewhere (e.g. Benowitz and Burbank [5] and Benowitz [4]). It should be noted that in discussing the risk to an elite and professional athlete arguably assumes a much lesser risk from underlying disease (e.g. cardiovascular, metabolic, etc.) and improved health profile than a general and clinical population, although this may not be the case, as, for example, even their lower risk increases during activity.

There is little doubt that when compared to tobacco use, medicamentous nicotine use alone (e.g. NRT) is considerably safer; however, there is some concern for chronic exposure to nicotine including cardiovascular disease, cancer, reproductive and perinatal disorders, and delayed wound-healing, whilst of course nicotine intoxication is also possible [4]. In humans nicotine has a relatively high toxicity (LD_50_ of 0.5–1 mg kg^−1^) when compared to other alkaloids such as caffeine (LD_50_ of 150–200 mg kg^−1^), although this has recently been questioned with Mayer [43] suggesting that the lower limit causing fatal outcomes is 6.5–13 mg kg^−1^ orally. Nicotine binding to nAChRs conveys different electrophysiological characteristics and agonist binding due to the subunit component varying by tissue; for example the α4β2 receptor mediates addiction, α3β4 receptors mediate cardiovascular responses, and α7 receptors activate the cholinergic immune system [5].

As a sympathomimetic stimulating catecholamine release, the cardiovascular effects of nicotine are most pronounced and act to alter cardiac rate, contractility and output, possibly alter coronary blood flow and may contribute to endothelial dysfunction that, in theory, precipitates acute ischaemic events [4, 5]; for an in-depth discussion, including both in vitro and non-human species models and other effects (e.g. arrhythmia), readers are directed towards other reviews (e.g. Benowitz and Burbank [5], Chagué et al. [11] and Haass and Kübler [24]). Nicotine induces lipolysis and could enhance insulin resistance and, therefore, promotes atherogenesis and type II diabetes [4, 5]. Although not a direct carcinogen, nicotine may be a tumour promoter through its actions on inhibiting apoptosis and promoting angiogenesis, whilst it is suspected that nicotine most prominently affects fetal neuroteratogenic effects [4, 5]. As a systemic venoconstrictor and through cutaneous vasoconstriction, the actions of nicotine in reducing blood flow in microvascular beds may contribute to impaired wound healing [4, 5]. Nicotine addiction is also co-morbid with mental health disorders, and nicotine potentially serves to medicate some psychiatric symptoms due to the similar neurochemical effects to some antidepressant medications [4].

It is important to consider whether acute nicotine exposure in vitro or in animal models can be generalised to human in vivo exposure, particularly over a prolonged period. Furthermore, as the majority of basic and clinical research has been gained from smokers and smoking (cessation), it is often difficult to ascertain the separate effects of nicotine and tobacco as tobacco users receive both concurrently. However, by carefully examining human experimental and epidemiological studies and the differences in response to smoked tobacco, ST (particularly low-nitrosamine snus), NRT as well as the impact of smoking cessation (in combination with NRT), a clouded picture becomes a little clearer, such that nicotine per se does not appear to increase the incidence or prevalence of hypertension, and is not a major contributor to a chronic inflammatory state or dyslipidemia with little or no increase of cardiovascular risk especially to those without an underlying cardiovascular disease or high risk [4, 5]. However, questions still remain about whether nicotine promotes cancer and it is not desirable to use nicotine during pregnancy, whereas long-term nicotine gum use has demonstrated a dose-response relationship between insulin resistance and hyperinsulinaemia [4, 5].

## Conclusions and Recommendations for Future Research

The legal status of nicotine within the sporting arena is simple: it is not prohibited and is available over-the-counter in a plethora of products, with WADA currently (only) monitoring its use and, to the author’s knowledge, no governing bodies contradicting this stance, although some do prohibit the use of tobacco-derived products (e.g. Féderation Française de Ski) that could encourage other means of nicotine consumption. Within the elite/professional athlete environment use of nicotine is high with most documented (ab)use in baseball, ice-hockey and other strength-based and winter sports, with team sport athletes using nicotine the most, and endurance athletes the least. As a cholinergic agonist nicotine’s predominant modes of action include a psychostimulatory effect through activating CNS reward pathways and a sympathoadrenal effect via the release of the catecholamines. However, the current (limited) evidence base for any performance-enhancing effect is weak as the majority of studies demonstrate no effect (ergogenic or ergolytic) on muscular, endurance or high-intensity exercise performance. Nevertheless, given the low number of studies that have assessed any consequence of nicotine on athletic performance and the notable limitations of those investigations reported herein, a concerted future research effort is required to sufficiently determine the extent of nicotine’s influence on performance, athlete health and wellbeing, and any argument for/against a change in its sporting legal status.

In order to better interpret future results, hallmarks of rigorous experimental design such as inclusion of a double-blind, placebo-control protocol with manipulation check are preferable and encouraged. To date few studies have sufficiently considered criterion validity of their laboratory performance tests or how expert performers (cf. recreationally active or competitive) might respond to nicotine, while obvious gaps in the literature point to recruitment of female athletes and a better understanding of how tolerant users are influenced through withdrawal (and re-introduction) and naïve users are influenced through (partial) desensitization. How environmental factors (e.g. extreme ambient temperature) and diet interact with nicotine supplementation also warrant investigation as, for example, intense or prolonged exercise in a hot environment may interact with nicotine’s known effects as a cutaneous vasoconstrictor and could place the athlete at greater risk of developing a heat illness. Peri-exercise meals (timing and composition) and consumption of certain ingredients that are widely used (e.g. menthol, grapefruit) will likely affect the rate of nicotine clearance and the maximal effect observed, and thus experimental testing should consider what, when and how athletes prepare for competition. It is likely that women, especially those taking oestrogen-containing oral contraception, will respond differently than men. A promising area to pursue concerns the various NRTs and their delivery of nicotine and resultant side effects, in an attempt to minimise these and maximise absorption. Finally, a challenging yet worthwhile avenue would be to address whether chronic use results in addiction and the effects on mental and physical health and wellbeing, especially as the use of nicotine during and in recovery from exercise may adversely affect athlete health in a synergistic (hyper-additive) manner.
